# A set of vectors for introduction of antibiotic resistance genes by *in vitro *Cre-mediated recombination

**DOI:** 10.1186/1756-0500-1-135

**Published:** 2008-12-23

**Authors:** Petr V Dmitriev, Yegor S Vassetzky

**Affiliations:** 1 Université Paris-Sud 11 CNRS UMR 8126 « Interactions moléculaires et cancer », Institut de Cancérologie Gustave-Roussy, F-94805 Villejuif cedex, France

## Abstract

**Background:**

Introduction of new antibiotic resistance genes in the plasmids of interest is a frequent task in molecular cloning practice. Classical approaches involving digestion with restriction endonucleases and ligation are time-consuming.

**Findings:**

We have created a set of insertion vectors (pINS) carrying genes that provide resistance to various antibiotics (puromycin, blasticidin and G418) and containing a loxP site. Each vector (pINS-Puro, pINS-Blast or pINS-Neo) contains either a chloramphenicol or a kanamycin resistance gene and is unable to replicate in most *E. coli *strains as it contains a conditional R6Kγ replication origin. Introduction of the antibiotic resistance genes into the vector of interest is achieved by Cre-mediated recombination between the replication-incompetent pINS and a replication-competent target vector. The recombination mix is then transformed into *E. coli *and selected by the resistance marker (kanamycin or chloramphenicol) present in pINS, which allows to recover the recombinant plasmids with 100% efficiency.

**Conclusion:**

Here we propose a simple strategy that allows to introduce various antibiotic-resistance genes into any plasmid containing a replication origin, an ampicillin resistance gene and a loxP site.

## Background

Antibiotics blasticidin S, puromycin and G418 are frequently used for selection of stably transfected mammalian cell lines [1]. For this purpose plasmid expressing a gene of interest may be cotransfected with a plasmid containing a convenient antibiotic resistance gene [2,3]. Alternatively the antibiotic resistance gene and the gene of interest can be combined in one plasmid [4].

Unfortunately, the choice of several antibiotic resistance markers is available only for few types of expression vectors (for example, pcDNA3.1 vector series, Invitrogen) [5]. That is why usually researchers have to introduce new antibiotic resistance genes into the original vector. In this case, the cloning strategy may be complicated by the absence of the unique and convenient restriction sites in the plasmids containing long inserts.

**Figure 1:**
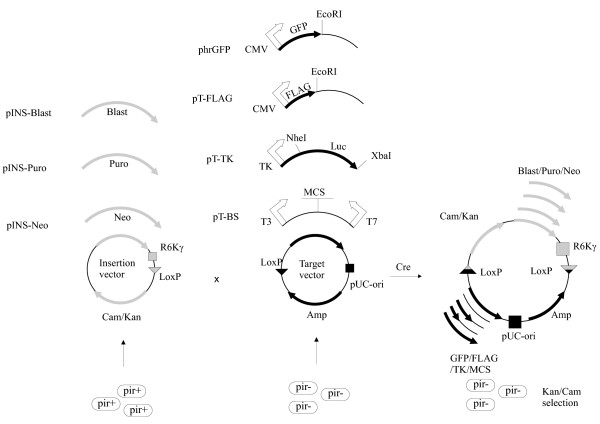
**General outline of the antibiotic genes introduction via recombination**. pINS plasmids (pINS-Blast, pINS-Puro and pINS-Neo) produced in the *pir+ E. coli *strains can be integrated via Cre-mediated recombination into any of the target vectors (phrGFP, pT-FLAG, pT-TK and pT-BS) produced in the *pir- E. coli *strains. The recombination mix is transformed into the *pir- E. coli *strain and the recombinant plasmid is selected by Kan or Cam markers provided by pINS vector.

**Figure 2:**
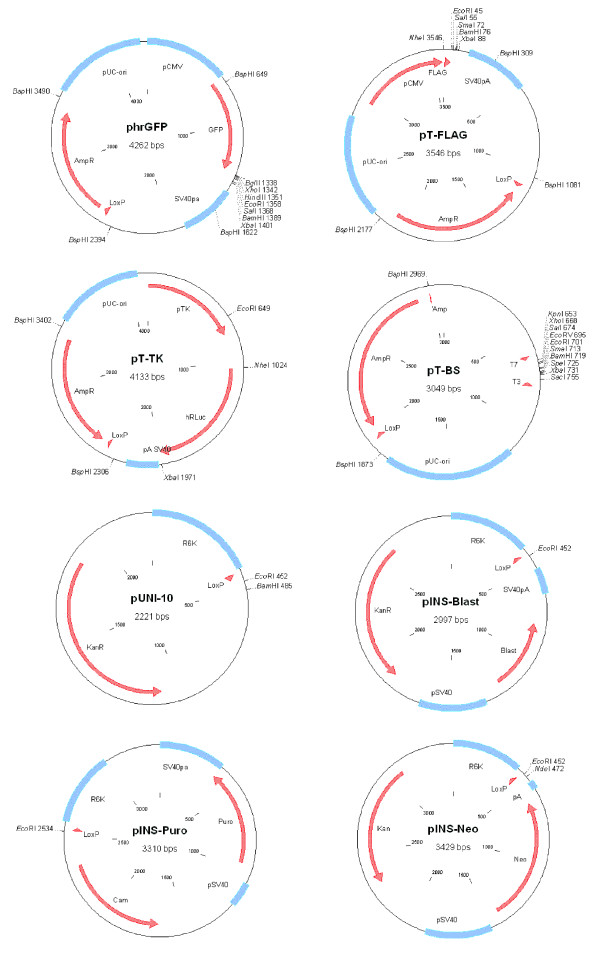
**Maps of Target (phRGFP, pT-FLAG, pT-TK and pT-BS) and Insertion (pINS-Blast, pINS-Puro and pINS-Neo) vectors**. pUNI-10 vector used as a backbone for cloning of the antibiotic resistance genes (Blast, Puro and Neo) is also shown. Only relevant restriction sites are shown. Detailed maps are available upon request.

Here we propose to introduce the antibiotic resistance genes using recombination (Fig. 1). We have created several insertion vectors (pINS-Puro, pINS-Neo, pINS-Blast) containing the **pac **(puromycin-N-acetyl transferase) [6,7], **aph **(aminoglycoside phosphotransferase) [8,9] and **bsd **(blasticidin S deaminase) [10] genes that provide resistance to puromycin, G418 (G418 is an aminoglycoside, similar in structure to neomycin) and blasticidin S respectively (Fig 2). pINS vectors can be introduced via Cre-recombination [11] into several commercially available target vectors containing the LoxP sites, for example phrGFP vector (Stratagene). In addition we created several new target vectors: pT-FLAG, pT-BS and pT-TK (Fig. 2).

### Construction of the insertion vectors pINS

We have used the backbone of the pUNI-10 plasmid [12,13] (Fig 1, 2) for construction of the insertion vectors pINS-Puro, pINS-Neo and pINS-Blast. pUNI-10 contains the R6Kγ origin of replication [14,15] and the LoxP site [11] recognized by Cre recombinase [16]. R6Kγ origin is active only in *E. coli *strains expressing the π-protein encoded by the ***pir ***gene. Cloning and production of the pINS plasmids was performed in the *pir+ E. coli *strain BW23474 expressing the mutant form of the π-protein (pir-116) that allows to maintain a plasmid with the R6Kγ origin at a high copy number [17,13].

Thus the pINS vectors contain four principal elements:

-R6Kγ origin of replication;

-LoxP site required for Cre-mediated recombination with target vector;

-Genes coding for either chloramphenicol acetyl transferase [18] or aminophosphotransferase [19] providing the resistance to the antibiotics chloramphenicol (Cam) or kanamycin (Kan), respectively. These genes are required for the selection of the recombinant constructs in *E. coli*;

- Genes coding for either **pac **(puromycin-N-acetyl transferase), **aph **(aminoglycoside phosphotransferase) or **bsd **(blasticidin S deaminase) controlled by the SV40 promoter. These genes provide mammalian cells with the resistance to puromycin, G418, or blasticidin S.

Conventional *E. coli *strains (XL-1 Blue, DH5α, JM-109 etc.) are *pir- *and cannot maintain the pINS plasmid. In contrast, the products of *in vitro *recombination between the pINS plasmid and the target vector can successfully replicate in the *pir- *strains due to the presence of the active origin of replication provided by the target vector. The selection of the recombinant plasmids is achieved by the markers Kan or Cam provided by pINS plasmid. This selection procedure allows to achieve 100% yield of recombinant plasmids (Fig. 1).

### Construction of the target vectors

Target vectors compatible with our pINS plasmids must contain only three necessary elements (Fig. 1):

- the LoxP site;

- An origin of replication active in the *pir- E. coli *strain, for example, pUC-origin [20];

- An appropriate antibiotic resistance gene, for example beta-lactamase (bla) [21] providing resistance to ampicillin (Amp).

We have modified several commercially available plasmids (phRL-TK (Promega) and pBluescriptII (Stratagene) by introduction of the LoxP sites resulting in the target vectors pT-TK and pT-BS respectively (Fig 2).

pT-TK vector contains the *Renilla *luciferase gene under control of the herpes simplex virus thymidine kinase promoter (TK) [22]. pT-TK vector can be used for the expression of a gene of interest at the levels that are 10-20 times lower than produced by the CMV promoter at least in some types of mammalian cells (HeLa, NIH 3T3 [23] and MEF [23]). For this purpose, the luciferase gene has to be cut out by NheI and XbaI and replaced by the gene of interest. Alternatively, any other vector can be used as a target vector in our system if upgraded by insertion of the LoxP sites as described [12].

pT-BS vector contains the convenient pBluescriptII polylinker [24] suitable for cloning of the expression modules containing a gene of interest under the control of appropriate promoter.

We have also used the commercially available target vector phrGFP (Stratagene) already containing the LoxP site. We have also created a pT-FLAG vector by replacing the GFP via FLAG-tag in the phrGFP vector (Fig 2).

pT-FLAG vector is coding for the FLAG-tag (DYKDDDDK) [25] and the cytomegalovirus promoter (CMV) [26]. It is suitable for cloning and expression of proteins with the N-terminal FLAG-tag.

All target vectors were cloned and produced in the XL-1 Blue strain (*pir*-).

### Introduction of an antibiotic resistance gene in the target vectors by *in vitro *recombination

We have performed *in vitro *recombination between the pINS and the target vectors using Cre-recombinase. We have transformed the *pir- *and *pir+ E. coli *strains (XL-1 Blue and BW23474 respectively) with the reaction mixture in order to test the efficiency of the reaction and selected the transformants using either kanamycin, chloramphenicol or ampicillin.

**Figure 3:**
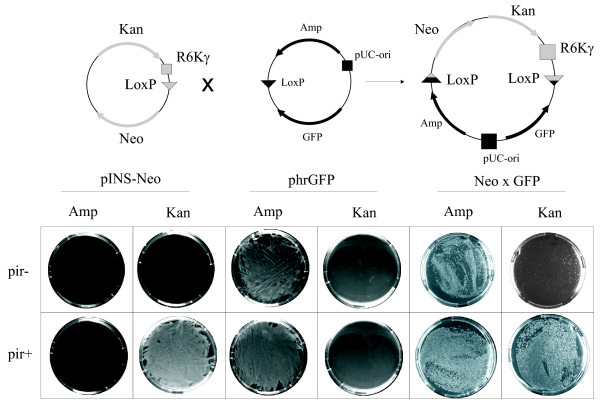
**Upper part. Schematic representation of the Cre-mediated recombination between Insertion vector pINS-Neo and Target vector phrGFP**. **Lower part**. XL1 Blue (*pir+*) and BW23474 (*pir-*) *E. coli *strains were transformed by the pINS-Puro, phrGFP vectors (50 ng each) and the recombination mix (Neo × GFP). 1/10^th ^of the transformed cells was selected on the LB plates containing either ampicillin or kanamycin. Insertion vector pINS-Neo containing R6Kγ can transform *pir+ *but not *pir- *strain and produces kanamycin-resistant colonies. Target vector phrGFP containing pUC-origin can transform *pir+ *as well as *pir- *strain and produces ampicillin-resistant colonies. Recombination mix contains initial vectors as well as the product of recombination and thus can transform both *pir- *and *pir+ *strains and produce ampicillin and kanamycin-resistant colonies. Only the recombination product containing both pUC-origin and Kan-marker can produce kanamycin-resistant colonies of the *pir- *strain.

Recombination mix contains the product of recombination (pINS × target vector) as well as the initial pINS and target vectors that did not take part in the reaction (Fig. 3). Recombination mix produced ampicillin-resistant colonies in cases of *pir- *and *pir+ *strains due to the presence of the initial target vector (Amp). The *pir+ *strain transformed by the recombination mix also produced kanamycin or chloramphenicol-resistant colonies due to the presence of the initial pINS vector (Can or Kan). In contrast, we have observed much fewer kanamycin- or chloramphenicol-resistant colonies in the *pir- *strain transformed by the recombination mix. These colonies only appear if the cells receive replication-competent product of recombination containing the kanamycin/chloramphenicol resistance gene (pINS × target vector). Alternatively, these colonies could appear from the contaminants of the initial plasmids.

In order to test the purity of our plasmid preparations, we have also transformed the pINS vectors and the target vectors into both *pir- *and *pir+ *strains and selected the transformants using either kanamycin/chloramphenicol or ampicillin (Fig. 3 and data not shown). As expected, the pINS vectors did not transform the *pir- *strain. In contrast, the *pir+ *strain transformed by the pINS vector can grow on either kanamycin or chloramphenicol, but not on ampicillin. The target vector transformed both XL1-Blue (*pir-*) and BW23474 (*pir+*) strains since the activity of the pUC origin of replication did not depend on the presence of the *pir *gene and produced the ampicillin-resistant, but neither kanamycin- nor chloramphenicol-resistant colonies. This confirmed the purity of the initial plasmids.

We calculated the yield of recombination (0.02%) by counting the kanamycin-resistant colonies of the *pir- *strain transformed by the recombination mix and taking into account the transformation efficiency (2.2 × 10^8 colonies/mkg DNA) (Fig. 3, and data not shown).

**Figure 4:**
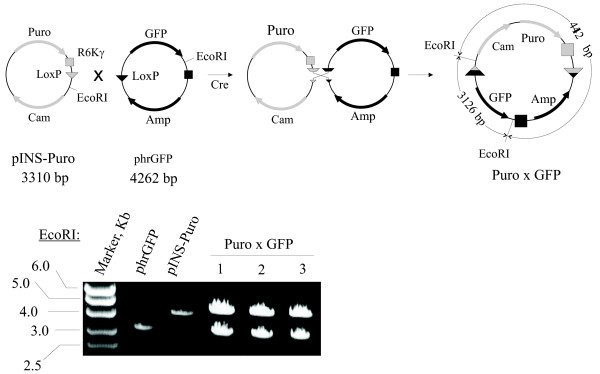
**pINS plasmids are introduced always in the same orientation**. **Upper part**. Schematic representation of the Cre-mediated recombination between Insertion vector pINS-Puro and Target vector phrGFP. Recombination intermediate and the sizes of the plasmids are shown. **Lower part**. Orientation of the pINS-Puro insert in the product of recombination (Puro × GFP) was analyzed by EcoRI. The sizes of the digestion products were analyzed on the agarose gel. We observed the fragments specific to only one orientation of the pINS-Puro in the product of recombination. This orientation is determined by the orientation of loxP sites.

In order to test the integrity of the recombination product, we have picked either kanamycin- or chloramphenicol-resistant colonies, isolated plasmid DNA and digested it with an appropriate restriction enzyme. We used EcoRI in case of recombination between pINS-Puro and phrGFP. All colonies gave the restriction pattern expected for the product of recombination, thus efficiency of the resistance marker introduction is close to 100% (Fig 4 and data not shown). Moreover, due to the directional nature of the LoxP sites, integration occurs in only one orientation depending on the orientation of the LoxP sites. This feature makes the population of recombinant vectors highly homogenous (Fig. 4 and data not shown).

**Figure 5:**
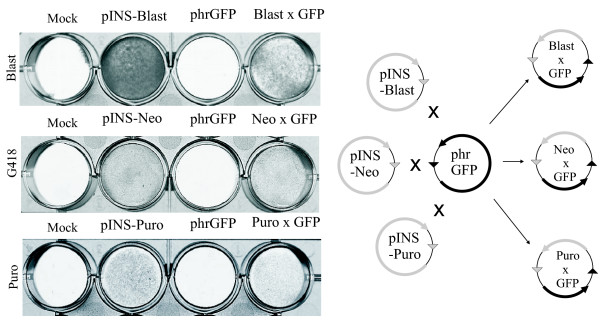
**Functionality of Blast, Puro and G418-resistance genes from pINS plasmids is preserved in the products of recombination**. **Left part**. Colony forming assay. HeLa cells transfected by 1 mkg of indicated plasmids were selected by either blasticidin S, puromycin or G418. After completion of selection the cells were stained by methylene blue. Insertion vectors pINS-Blast, pINS-Neo and pINS-Puro and the products of their recombination with Target vector phrGFP (Blast × GFP, Neo × GFP and Puro × GFP) provide HeLa cells with the resistance to blasticidin S, G418 or puromycin respectively. **Right part**. Schematic representation of the Cre-mediated recombination between Insertion vectors pINS-Blast, pINS-Neo and pINS-Puro and Target vector phrGFP.

Next we have verified whether the function of Puro-, Blast- or G418-resistance genes from the pINS-plasmids and the gene of interest from the target vector is preserved in the product of recombination. For this purpose we have performed the recombination between each of the three insertion vectors (pINS-Neo, pINS-Puro and pINS-Blast) and the target vector phrGFP. Then we have transformed the recombination mix into the *pir- *strain and selected the cells containing the product of recombination by growing them on the kanamycin- or chloramphenicol-containing plates. Then we transfected the initial plasmids and the product of recombination (Neo × GFP, Puro × GFP and Blast × GFP) into HeLa cells and analyzed their resistance to either puromycin, blasticidin S or G418. As expected, only pINS vectors and the products of recombination provided the HeLa cells with the resistance against blasticidin S, puromycin and G418 (Fig 5).

**Figure 6:**
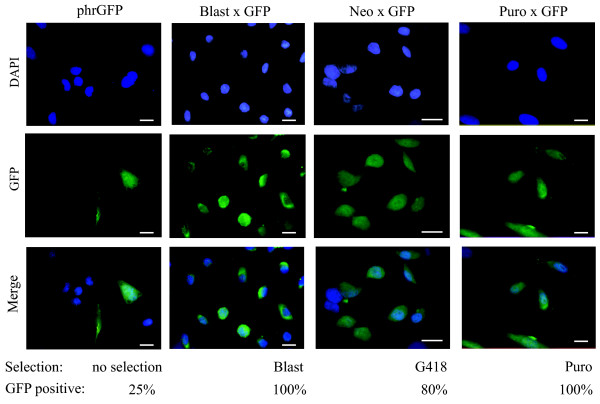
**Functionality of GFP gene from phRGFP plasmid is preserved in the products of recombination with pINS-Puro, pINS-Neo or pINS-Blast plasmids**. HeLa cells transfected by 1 mkg of either Target vector phrGFP or the products of recombination (Blast × GFP, Neo × GFP and Puro × GFP). Cells transfected by the recombination products were selected by either blasticidin S, G418 or puromycin and stained by DAPI. Expression of the GFP was analyzed under the microscope. In case of transient transfection by phrGFP vector we usually observed 25% GFP positive cells. In contrast we observed that 80–100% of the cells transfected by the products of recombination and selected by the corresponding antibiotics are GFP positive. Scale bar: 20 μm.

Then HeLa cells resistant to the antibiotics were inspected under the microscope for the expression of GFP. Only cells transfected by the recombination products were GFP-positive. Moreover, the proportion of the GFP-positive cells was considerably higher than in the case of transient transfection by the phrGFP plasmid (Fig 6). We conclude that our recombination procedure can "safely" merge the antibiotic resistance gene and the gene of interest in one plasmid.

## Discussion

Researchers working with the vectors suitable for expression in mammalian cells often meet with the puzzle of how to quickly introduce or switch the antibiotic resistance gene in the vector of interest. In this paper we describe the pINS vectors suitable for introduction of antibiotic resistance genes in any plasmid of interest containing the LoxP site by Cre-mediated recombination. We cloned the genes providing resistance to three frequently used antibiotics (puromycin, blasticidin S and G418) in the pUNI-10 plasmid [12]. The plasmid pUNI-10 contains the R6Kγ origin of replication which is inactive in majority of *E. coli *strains routinely used for cloning (they are *pir-*). The plasmid resulting from recombination between the pINS and a target vectors acquires a gene of interest (provided by the target vector) and resistance to an antibiotic (puromycin, blasticidin S or G418) (provided by pINS vector). The principle of selection in based on the acquisition of the functional origin of replication (provided by the target vector) as well as the marker providing resistance to kanamycin or chloramphenicol (provided by the pINS vector). Combination of these features makes it possible to select the recombinant plasmids by transforming the recombination mix into a *pir- E. coli *strain and selecting the kanamycin- or chloramphenicol-positive colonies (Fig. 1). These two features are necessary and sufficient for selection of the recombinant plasmids. Utilization of two antibiotics (chloraphenicol/kanamycin+ampicillin) does not provide any further enhancement to the procedure.

Our method is similar to the procedure used in the existing pExchanger system (Stratagene). pExchanger system relies on integration of the linear fragments coding for the antibiotic resistance genes and selection marker (Kan or Cam) into the target vector using Cre-recombination. Linear fragments can't replicate by themselves and thus can't efficiently transform *E. coli*. Presumably only the colonies containing the recombinant plasmids can be selected by the markers encoded in the linear fragments. In contrast to pExchanger system, we propose to use the replication-deficient circular pINS plasmids that can be easily produced in the user laboratory and thus allow the user to cut the cost of the cloning.

The Insertion vectors pINS-Puro, pINS-Neo and pINS-Blast are compatible with the numerous target vectors already present on the market (for example, pExchange core or phrGFP vector families, Stratagene). In addition, virtually any vector of interest can be converted into a target vector by simple introduction of the LoxP site.

## Methods

### pINS plasmids

In order to obtain the pINS-Blast vector, the pcDNA6/TR vector (Invitrogen, Carlsbad, CA, USA) was digested by XmnI and SalI and the fragment containing blasticidin S resistance gene was ligated with the pUNI-10 vector (kindly provided by Dr. Stephen Elledge) digested by EcoRV and SalI (these and other restriction enzymes were purchased from Fermentas, Vilnius, Lithuania) using T4 ligase (Fermentas). The product of ligation was transformed by electroporation into the strain BW23474 (Δlac-169 rpoS(Am) robA1 creC510 hsdR514 endA recA1 uidA (ΔMluI)::pir-116) that was kindly provided by Dr. Stephen Elledge, and selected using kanamycin. The resulting plasmid was named pINS-Blast.

In order to obtain the pINS-Puro vector, the pPur plasmid (Clontech, Terra Bella, CA, USA) was digested by NdeI, blunt-ended using Klenow fragment (Fermentas), then digested by BamHI and ligated with the fragment of pACYC-184 plasmid [27] containing Cam resistance gene (pACYC-184 was purchased from Fermentas). In order to obtain this fragment we first digested pACYC-184 by BclI, treated it with Klenow fragment, then digested with BamHI. The product of ligation was named pACYC-Puro. pACYC-Puro was digested by Bst1107I, BamHI, then blunt-ended with Klenow fragment. The fragment containing chloramphenicol and puromycin resistance genes was ligated with pUNI-10 vector digested EcoRI and BglII and blunt-ended with Klenow fragment. The product of ligation was transformed via electroporation in BW23474 strain and selected via chloramphenicol. The resulting plasmid was named pINS-Puro.

In order to obtain the pINS-Neo vector, pCINeo plasmid (Promega, Madison, WI, USA) was digested by BamHI and the fragment containing pUC-origin, ampicillin and neomycin resistance genes was ligated to BamHI-linearized pUNI-10 resulting in the plasmid "3490". Plasmid "3490" was digested by XbaI and self-ligated in order to remove ampicillin resistance gene and pUC-origin of replication. The product of ligation was transformed into the BW23474 strain via electroporation and selected using kanamycin. The resulting plasmid was named pINS-Neo.

### Target vectors

In order to prepare the pT-FLAG vector, we digested phrGFP vector (Stratagene) by NheI and EcoRI and replaced the GFP ORF by the oligonucleotide duplex encoding the FLAG-tag (oligo1: 5'-CTAGCCCATGGATTACAAAGACGATGACGATAAACCTAGCTTCG; oligo2: 5'-AATTCGAAGCTAGGTTTATCGTCATCGTCTTTGTAATCCATGGG) (Sigma, St. Louis, MO, USA). Then LoxP-site coupled to ampicillin resistance gene was isolated from pT-FLAG plasmid by BspHI digestion and cloned into phRL-TK (Promega, Madison, WI, USA) or pBlueScriptII (Stratagene) digested by BspHI. The resulting plasmids were named pT-TK and pT-BS respectively.

### *In vitro *Cre recombination

500 ng of pINS vector was mixed with 500 ng of target vector, 5 units of Cre recombinase (Stratagene, La Jolla, CA, USA), and a buffer recommended by manufacturer in 10 mkl reaction volume. The reaction was incubated for 30 min at 37°C, then Cre recombinase was heat-inactivated at 65°C for 20 min. The recombination mix was transformed by electroporation into XL1-Blue *E. coli *strain (*recA1 endA1 gyrA96 thi-1 hsdR17 supE44 relA1 lac *[F' *proAB lacI*q*Z*Δ*M15 *Tn*10 *(Tetr)]) purchased from Stratagene and selected using either 34 mkg/ml chloramphenicol (Euromedex) or 50 mkg/ml kanamycin (Euromedex, Mundolsheim, France).

### Testing the performance of the constructs in HeLa

Dulbecco's modified Eagle's medium (DMEM #31885 Gibco, Carlsbad, CA, USA) supplemented with 10% heat-inactivated Millerium Fetal Bovine Serum (#BWSTS1810 VWR International, Fontenay-sous-Bois, France), 100 units/ml penicillin G and 100 mkg/ml streptomycin sulfate (Gibco) and 500 ng/ml Fungizone (Invitrogen) in the presence of 5% CO_2_. Cells were transfected by 1 mkg of plasmid DNA using JetPEI (Polyplus-transfection Inc., New York, NY, USA) according to the protocol provided by supplier. 24 hours after transfection media was replaced and the cells were either grown for another 24 hours (transient transfection) or selected with either 3 mg/ml puromycin (Sigma #P8833) for 3 days, 5 mg/ml blasticidin S (Sigma #15205) for 5 days or 1 mg/ml G418 (Sigma #G8168) for 10 days until the complete death of mock-transfected cells. Transfected cells were either stained by 1% methylene blue (Euromedex #A514) in 50% methanol or mounted on the slides, stained by 100 ng/ml DAPI (Sigma #D9542) and inspected under the fluorescent microscope Olympus AX70.

## Availability and requirements

Plasmids and their sequences are available upon request.

## Competing interests

The authors declare that they have no competing interests.

## Authors' contributions

P.D. designed and performed the experiments, Y.V designed the experiments and wrote the paper.
